# Medical and Philosophical Intelligence

**Published:** 1814-11

**Authors:** 


### MEDICAL AND PHILOSOPHICAL INTELLIGENCE.

AT a meeting of the *Westminster Medical Society*, held on Saturday, Oct. 22, Mr. HOWSHIP in the Chair, Mr. EARLE called the attention of the Society to the consideration of a disease affecting the extremity of the meatus urinarius of the female, consisting in a tumour of a florid red colour, composed apparently of a congeries of minute blood-vessels, somewhat resembling nævi materni, arising sometimes from a part, at others from the whole circumference of the passage. This complaint, he observed, was exquisitely painful on the least possible touch. He has seen five cases, in all of which caustic applications had been repeatedly and extensively employed, but without producing any permanent benefit, the granulations which threw off the sloughs taking on the same morbid sensibility. This led him to employ the scalpel in preference, from the expectation that the wound inflicted would heal without granulating. With this view, in a case which had existed for upwards of twenty years, he removed the whole circumference of the extremity of the meatus, and dissected out a considerable portion of the membrane, leaving the urethra. Some hæmorrhage ensued, which subsided with cold washes. The result fully answered his expectations; the wound healed, and no return of the complaint had since taken place. In this case the whole of the meatus urinarius was thickened and morbidly sensible, and the neck of the bladder painful and irritable. These symptoms are all much relieved, but not perfectly removed.

The same operation was afterwards performed in a similar case, of seven years standing, with equal success. In the second case, the disease was confined to the extremity, and consequently the relief obtained was more complete. He has a third case now under his care, which he intends to treat in the same manner. This last was formerly treated by caustic potass, with apparent, but only temporary, benefit.

Mr. WANT communicated two cases somewhat similar to the foregoing. In his patients the diseased alteration of structure was situated in the nymphæ and integuments of the clitoris, and consisted in an exquisitely painful thickening, accompanied with great discharge of mucus from the vagina. The colour of the parts was not, as in Mr. Earle's cases, florid, but had a livid erysipelatous hue. The treatment adopted was that of local depletion by leeches, which seemed to afford temporary relief, but was not sufficiently persevered in to ensure success, had it been attainable. One of the subjects of this disease, a patient of the Northern Dispensary, was discharged for irregularity while under cure; the other, on a proposal being made to remove the diseased parts by the scalpel, as the most speedy and certain remedy, withdrew herself, so that the result of neither case is known.

Mr. NEVILLE related a case of affection of the stomach consequent to the use of arsenic, and supposed to be occasioned by it.

A young woman, æt. 26, before marriage, rather irregular in her habits, was attended for some suspicious sores on the tibia, considered to be pseudo syphilis, which resisted a variety of applications. Mercury was administered in alterative quantity without benefit. Arsenic and Sarsaparilla were then combined, and exhibited with great advantage. After three weeks, symptoms of considerable debility suddenly supervened, followed by violent pain in the head and stomach, with vomiting, and sensation of burning in the epigastric region. Bleeding, with local blistering, subdued the symptoms. In three days they recurred, and were again controuled by the same remedies: they recurred once again, and were similarly remedied, notwithstanding the greatly debilitated condition of the woman. Since her recovery from the affection of the stomach, the symptoms for which arsenic was administered are rapidly getting well.

---

Dr. CALCAGNO has published the result of his further experience on the internal use of Charcoal, in a letter "Sull' uso interno del Carbon di Legno," &c. wherein he relates his having cured a double tertian in the person of Giuseppe Bellavia, a patient in his hospital, by administering the powder of charcoal in the dose of a drachm every hour during the intermissions, but without the addition of an acid, which he formerly thought necessary. The fever did not return after two ounces of the powder had been administered.

From the 23d September to the 13th October, he had an opportunity of employing it in five other cases, in four of which the remedy succeeded by the time two ounces and a half had been given; but in the fifth, the fever not being removed after the exhibition of three ounces, he judged it prudent to have recourse to bark, which prevented the following paroxysm, although but half an ounce of the latter had been prescribed. The period of this paroxysm being over, he again ordered the charcoal, and the patient continued well.

Maccadino, his pupil, on returning to Calatafimi, his native place, in the month of August, succeeded in curing eight cases of intermittent fever with the same remedy; and Signior Francesco Buscarelli, surgeon, following his example, found it useful in four other cases of the like nature.

"In the district of Aliminusa, (says Dr. Calcagno,) I know that the Journal in which the discovery of the effects of charcoal was first announced had scarcely arrived, when the practice became general, the inhabitants adopting it (the charcoal) as well without the prescription of the physician as with it; and these write to me in a letter, bearing date the 23d December, 'That the patients labouring ing under intermittent fevers that have been cured at this place by means of the powder of charcoal amount to a hundred and five; that each has not taken more than two ounces of the charcoal; and that the charcoal made use of was that prepared from the *stinco*.'" Dr. Nicosia also employed it in seventeen other cases in the district of Nissoria, according to the testimony of Dr. Bursa of Palermo.

But, however assiduous the Sicilians may have been in taking advantage of a remedy they very much required, they have not been without rivals in many of the British practitioners, under the auspices and control of Dr. Borland, inspector of army hospitals. Mr. Mackesy, surgeon of the 62d regiment, has permitted to be published in Italian seven interesting cases, in which the charcoal was successfully employed by him; but, as these cases will probably be published in England, I shall not enter into particulars respecting them. Mr. Tully, surgeon, 35th regiment, in a report made to the inspector of hospitals, dated Zante, November 12, 1813, states, that he has found the remedy particularly successful in remittent and intermittent fevers of that island; and that its administration, in more than thirty cases, principally intermittents, has been attended with the most happy effects. "In obstinate cases of diarrhœa, and in the last stages of dysentery," he adds, "I have seen that the charcoal, in doses from 15 to 20 grains given three or four times a-day, is a most excellent remedy."

---

It has generally been supposed that vomiting is produced by the action of the muscular fibres of the stomach. Bell, Chirac, and Duverney, maintained the contrary; but the arguments adduced were insufficient to establish their opinions. A German physiologist, Wepfer, in some experiments made for the purpose of ascertaining the fact, having introduced poison into the stomach of an animal, excited spasmodic action, which he mistook for the contraction of vomiting. No doubt existed on the subject, until M. Magendie made his experiments on dogs, in which vomiting was excited by emetic tartar injected into the veins.

It is important to remark in these experiments, that, when the emetic is employed in this manner, vomiting is excited in a few minutes; whereas, when introduced into the stomach, the effect is often not produced in an hour. When nausea had taken place, the finger was introduced into a wound of the abdomen, and was strongly compressed by the diaphragm and abdominal muscles, but no movement was felt in the stomach. A portion of this viscus being withdrawn from the wound, the vomiting was excited without any contraction observed in it. When the stomach was drawn from the abdomen, nausea and efforts to vomit continued, but vomiting was not effected.

If the stomach thus situated is compressed, its contents are certainly evacuated, but purely by *mechanical pressure*. M. Magendie detached the whole of the anterior position of the abdominal muscles in a dog. The effort to vomit continued, but without effect; and the peritoneum was ruptured by it in several places.

These

These experiments appeared decisive; but M. Magendie made another still more conclusive and extraordinary. He removed the stomach of a large dog, taking at the same time the necessary precautions to avoid hæmorrhage, and substituted in its place a pig's bladder, which was fastened to the œsophagus by means of a canula. The external wound being sewed up, vomiting was produced, as well as if the stomach had been in its place. It is evident from these experiments, that the stomach is quite passive during vomiting, and that it is not by an action upon the stomach only that emetic medicines produce their effects.—This is a transcript from the Gazette de Santé, which we present to our readers not from its actual novelty, but because, like many other undoubted facts, it has slept, as far as regards the mass of the professional public, for nearly one hundred years. M. Magendie will excuse us for denying the assertion, that the arguments of Chirac and Duverney were insufficient to establish the facts; they seemed to us to be conclusive; but, at the time the experiments were shewn to the French Academy, that learned body being engaged in other speculations, the subject was laid aside, to be confirmed by a repetition or extension of the experiments at their leisure.

---

The Italian surgeons seem to be entirely ignorant of the property which the ligature on an artery possesses of rupturing its inner coat. In this country it has been undeniably demonstrated, that the permanent obliteration of these vessels can only be affected by the adhesion of the surfaces thus divided. Hence, it was stated by Dr. Jones, that the application of a ligature, with a degree of sufficient force to produce the division of the internal coat, would obliterate its cavity, even if it were immediately withdrawn. An Italian professor, Asselini, who is now in this country, attended at St. George's, for the purpose of witnessing an experiment upon the fæmoral artery of a dead subject. The ligature was applied by Mr. Brodie, and a portion of the artery removed, when, to the astonishment of the foreigner, the inner coat was most clearly divided. The establishment of this fact is of the greatest importance to surgical science, especially as it regards the operation for aneurism. Until lately, it was the practice of the operator (fearing the division of the coats) to include a small portion of some extraneous body between the ligature and the artery; thus in effect preventing that which is of the utmost consequence to the success of the operation, and without which secondary hæmorrhagy so frequently occurs.

---

M. Carron, physician at Annecy, has published observations on the use of opium in the dry gangrene, which frequently attacks the toes. Pott was the first who made known the efficacy of opium in the dry gangrene of the lower extremities; his experience led him to believe that it arose from a high state of irritability in the feet, and that opium was the most proper to allay it. M. Carron related three cases, which seemingly confirmed this doctrine. The first was a man, 65 years of age, who had been threatened with with an attack of hemiplegia; dry gangrene supervened, and opium given in the dose of two grains abated the pains, but induced drowsiness. Spread, however, on a poultice, it produced no bad effect on the constitution, but quickly stopped the gangrene. In the second case, the gangrene had made dreadful progress, and was accompanied with violent pains: but, as the subject was a man 84 years of age, and attacked at the same time with typhus gravior, opium did not so immediately relieve the pains. Every application in this case failed. There was delirium, insensibility, constipation, dry black furred tongue, the toes entirely sphacelated, the leg and thigh covered with phlyctenæ, which destroyed the patient. The third was a man 60 years of age, who for some months had indications of gangrene, which made slow progress, but was very painful. Two months afterwards, he ventured upon taking opium internally, which immediately relieved the pain, and stopped the gangrene; the sphacelated parts separated, and the wound cicatrized; but the circulation is extremely languid, and the patient continues to feel a sensation of coldness in the feet.

---

Mr. Kerrison has published a case of hydrophobia, which, like most of its predecessors, terminated fatally.

The day after the accident caustic was applied to the part bitten. Five weeks after (July 7), upon the first appearance of indisposition, a cathartic medicine was prescribed; at this time no abhorrence of liquids was present.

Friday, July 8th, 10 A.M.—The boy had not drank all the draught, and was sick last night after taking it; the mother believes he vomitted all he had taken. One stool however had been procured in the night. He had shewn great difficulty in drinking his tea at breakfast. There was dulness of the eyes, and languor of the countenance, with a furred tongue and hurried pulse.

I now suspected the boy was under the influence of hydrophobia; but as it was considered necessary to prove its existence, before any intimation of the suspicion was conveyed to the mother, or any language used which could alarm the boy: a goblet of spring water was brought, and he was requested to drink. He took hold of the goblet, and attempted to drink; but, as soon as the glass approached his lips, a convulsive motion of the muscles of the fauces and throat prevented him from swallowing a drop of the liquid: he renewed his efforts, but without success. He was nevertheless requested to make a third attempt, and now got down about a table spoonful.

After having heard the preceding account, a goblet was procured; and whilst the water was gushing from a reservoir in the room into the glass, two distinct convulsions of the muscles of deglutition were remarked, corresponding in time with two strokes of the pump-handle by which the water was raised. When the boy attempted to drink he shuddered, and threw back his head several times; and the vessel was then taken from him, without his having tasted its contents. He was perfectly free from delirium, and seemed to be of a placid disposition, having done, without reluctance, every thing that he was ordered to do. He walked home with his mother.

Fourteen ounces of blood were directly drawn from the arm; and an enema, containing one hundred drops of Tinct. Opii in four ounces of water, was administered immediately afterwards. A powder, consisting of one grain of Argenti Nitras and three grains of musk, mixed with six of sugar, was ordered to be repeated every six hours; a dram of Ung. Hydr. fort. to be rubbed in on the abdomen, and repeated every three hours; and a lotion, made of Sp. Æth. Comp. f ℥ i. Acid. Acet. f ℥ iii. and Aq. Distillat. Oi., to be applied to the anterior part of the throat by a piece of folded linen, and kept constantly wet.

Five P.M.—The clyster is still within the rectum. The boy has been dozing at intervals, and he is now asleep. There is a constant and equal convulsive action of the muscles under the chin and about the *pomum adami*. On feeling his hand, he awaked with surprise; but, in a moment, was quite collected. The pulse is 108, and moderately strong. The mother says he has taken some sago since one o'clock, and drank milk and water. On presenting a wine-glass of water, at this time, he drank a little of it with less difficulty than at eleven o'clock.

Half-past six P.M.—An increase of pulse and an aggravation of the spasmodic symptoms being perceived, ten ounces of blood were taken, and another enema, containing 180 drops of Tinct. Opii in two ounces of water, injected. The powders, ointment, and lotion, had been continued as directed. A noisy breathing, whilst he was dozing, was now perceptible, and the tongue was involuntarily, but not violently, acted on by the convulsive efforts of the muscles connected with its posterior part. This became visible during his slumbers, when the mouth was partly open; for the tongue was observed to be propelled and retracted in exact time with the motions perceived on the anterior part of the throat.

Ten P.M.—There has been much dozing since my last visit, with sudden starts; and there is now a confused state of perception when awake. The pulse is 110, but considerably weaker. It feels more like a sinking pulse in typhus, than the vibratory stroke of the artery in fever from local inflammation. Same medicines continued.

Saturday 9th, half-past 8 A.M.—My patient passed a restless night. The debility is much increased; the convulsive twitches are stronger; he lies almost constantly in a muttering slumber, and, when awake, is in a state of delirium. Some sago had been taken in the night. A cathartic powder, with calomel, was directed to be given.

Ten A.M.—The powder had produced a copious motion within an hour; there was now an unmeaning stare and wild motion of the eyes, alternately dropping into slumbers and starting, as if the child was under the influence of fear. Fifty drops of Tinct. Opii were given at this time, and forty more an hour afterwards.

Twelve at Noon.—There was no change of the symptoms, except that a secretion of frothy saliva was apparent, which seemed to give trouble, trouble as the boy put his fingers to his lips, and endeavoured to pull it from the mouth. The gums were inspected, but no indication of mercurial influence could be perceived. A large blister was ordered to be applied to the head.

Two P.M.—There are now violent contortions of the body and limbs, with perfect delirium, and the saliva is evidently inconvenient. He has not been able to swallow any thing since he took the sago, except the medicines.

Half-past three P.M.—The convulsions are very strong: the frothy saliva abundant; and the pulse irregular and sinking. Let twenty drops of Tinct. Opii be taken every half hour. One dose was immediately given with much difficulty. It was obvious that the disease was verging to its termination. A spasm very soon came on, which interrupted respiration for a short time, with a temporary cessation of the pulsation at the wrist. Respiration, however, returned, and the motion of the carotid arteries was visible; but, in about a quarter of an hour afterwards, a renewal of the spasm proved fatal.

The muscular contraction of the throat seemed at first to be lessened by the means employed, but it was soon found to be productive of great debility, without a corresponding decrease in the symptoms.

Mr. K. suggests the use of belladona, which it is generally known was recommended by Munnick as a *specific* for this complaint.—*Repos.*

---

Mr. KERRISON has given a case of Tic Douloureux, which seemed to be relieved by belladonna after the complete division of the nerve had been tried without effect. We hope it may be found equally successful in the hands of others.

One grain of the extract of belladonna was given in a pill, which was found to occasion vertigo, and great lassitude, with a peculiar and distressing dryness of the tongue and fauces; but the pain was removed. It returned the next day, and was again kept in check by smaller doses of the same medicine. A quarter of a grain of the extract was given three times a day, and increased to a third of a grain; then, a quarter of a grain morning and noon, and half a grain at night, sometimes omitting the medicines altogether for a day. The pain, whenever it returned, was as certainly removed by the medicine. In the course of three weeks, the disposition to the renewal of the *Tic* gradually subsided, and the remedy was consequently discontinued. No derangement of arterial action was perceived during this guarded use of the belladonna, nor were the bowels constipated. An augmentation of the dose was now and then attempted; but confused perception in a slight degree, and dryness of the mouth, always succeeded, although these effects subsided spontaneously in a few hours. The individual, with a mind of the highest order, and long habit of appreciating the effects of various medicines, was convinced of the power of this remedy over his complaint. Large doses of tincture of opium, and opium in substance, had previously been tried, without any good being obtained from them in this attack; and hemlock on a former occasion had also failed. The officinal solution of arsenic at that time, pushed to its full extent, had in some measure relieved, but not cured, the disease; but it had been discontinued from its deleterious influence on the general system.—*Repos.*

---

M. BECLARD, director of the anatomical works of the faculty of medicine in Paris, has published the case of a fœtus, the anterior lobes of whose brain passed out at the frontal suture, forming a tumour as large as the head. At the inferior nasal part of the frontal suture, were found two small extra bones, which were united to the palatine processes of the superior maxillary bones.

The same gentleman has also published a case of fœtus, the base of whose umbilical cord spread into a considerable sac, which enveloped the greatest part of the abdominal viscera and the thoracic viscera, the face, and part of the head. The heart, which was inverted, passed into the abdomen through an opening into the diaphragm, the apex adhering to the palate. This fœtus had also a voluminous hydro-encephalical hernia, and the feet were turned upside down.

---

DR. JOHN has analysed some vegetable and animal substances, which are published in a book written by the author. The first section of this work contains the analysis of the juice of the euphorbia cyparissias. It is composed of

| | |
|---|---|
| Water | 77 |
| Tartaric acid | Trace |
| Resin | 13·8 |
| Gum | 2·75 |
| Extractive | 2·75 |
| Albumen | 1·37 |
| Caoutchouc | 2·75 |
| Oil | Trace |
| | 100·42 |

The earthy parts of the euphorbia are composed of the carbonate, sulphate, and phosphate of lime.

The analysis of the asclepias syriaca furnished

| | |
|---|---|
| Resin | 26·50 |
| An elastic substance | 12·50 |
| Vegetable gluten | 4·00 |
| Tartaric acid and albumen | 53·00 |
| | 96 |

The plant, when incinerated, furnished carbonate of potash, phosphate of lime, phosphate of magnesia, silica, iron, and oxide of manganese.

The author next analysed an elastic scarlet-coloured substance which comes from the east through Turkey, and which is known by the name of caoutchouc of Thibet. This brilliant and globular matter serves for bracelets, ear-drops, rosaries, &c. to the Russian ladies ladies: and the substance which was usually considered as caoutchouc is only an indurated and oxidated red oil. Its colouring matter is analogous to that of stick lac.*

The fruit of the rus typhinum contains gallic acid at the first moment of its developement; but, as it advances to maturity, bitartrate of lime is formed; and, as soon as the circulation of the sap in it has ceased, acetic acid is formed. This leads to the suspicion that the acetic acid has been formed at the expense of the tartaric.

Galipot, according to our author, is composed of a volatile oil and a resin. According to other authors, galipot is nothing else than a very impure specimen of the resin called *elemi.*

*Analysis of Rocou.*—The rocou of commerce is a substance already altered by fermentation. The author procured grains of it, from which he obtained the following result:—Rocou contains an aroma, an acid, resin combined with the colouring matter, vegetable mucilage, fibrin, coloured extractive, and a peculiar matter which approaches to mucilage and extractive. This analysis explains the reason why an alkali is added to rocou when it is employed in dyeing. The alkali combines with the resin, and forms a soap which dissolves in water. The alkali acts likewise on the colouring matter, and renders it more lively.

The second section of Dr. John's work contains observations on diabetic urine. Though neither urea nor gelatine are to be found in diabetic urine, it notwithstanding contains azote. Diabetic urine is composed of sugar, animal gum, urate of potash, sulphate of potash, muriate of soda and ammonia, phosphate of magnesia, and phosphate of iron.

Some experiments on the excrements of butterflies lead the author to suspect that they contain uric acid.—*Ann. of Phil.*

---

M. Hildebrandt has recently made some curious experiments on the preservation of the flesh of animals in the gases. In a receiver of the capacity of three cubic inches, filled with very pure sulphurous acid gas, he introduced through mercury a piece of fresh beef: in a few minutes the meat had absorbed almost the whole gas, and the mercury filled the capacity of the receiver, except some air-bells, which were probably owing to the atmospheric air. The meat soon lost its natural red colour, and assumed that of boiled meat: it did not undergo any other apparent alterations, and the air in the bell-glass preserved its volume. At the end of seventy-six days, during which time the temperature had varied from 0 to 10° Reaumur, the meat had scarcely acquired any smell of sulphurous acid: it was harder and drier than roasted meat. After leaving it four days in the open air, it then became more compact without being putrefied, and did not perfectly change colour: it merely lost the weak smell of acid without acquiring any other.

A piece of ox beef was treated in the same way in the fluoric acid

---

* Bucholz has published an analysis of it in the first vol[illegible] Schweigger's Journal. He found it a concrete fixed oil [illegible] colour; but could not succeed in extracting from it [illegible] colouring matter, as announced by John.

gas, and the results were in every respect similar: the phenomena were only less visible, because the acid attacked the glass, and a thin coating of mercury was deposited on the meat.

Beef, deposited in a receiver filled with ammoniacal gas, exhibited alterations completely different: the absorption of the elastic fluid had taken place in it totally; the meat assumed a fine red colour nearly as in the nitrous gas, and preserved this fresh appearance for seventy-six days: it was much softer than in the foregoing experiment, without smell, and having the colour and consistence of fresh meat. When exposed four days to the open air it did not putrefy; it lost its red colour however, became brown, dried up, and was covered with a kind of varnish.

---

*On the formation of Pyrophorus, by John Gorham, M. D.*—In the first number of the Annals of Philosophy, Dr. Thompson, the editor, has inserted an extract from a letter of Professor Coxe of Philadelphia, in which it is mentioned, that a few drops of a solution of potass, added to the usual materials of pyrophorus, will greatly increase its susceptibility to spontaneous inflammation, on exposure to the air.

This fact respecting the addition of potass, I ascertained about eighteen months ago. Having made attempts to decompose the alkali by iron turnings, and having failed, as I supposed, from the want of a sufficiently intense heat, it occured to me that, as pyrophorus probably contains potassium, that metal might be obtained at a comparatively low temperature, by mixing with the iron and potass a quantity of the materials of which that combustible compound is formed. Accordingly about an ounce of the dried powder of alum and sugar, after they had been exposed to heat, was added to one ounce of clean iron filings, and half an ounce of dry caustic potass; this mixture was put into a gun barrel, arranged in the usual way for procuring potassium, and exposed for some time to a temperature approaching to a white heat. The experiment did not succeed, but the result was a very fine pyrophorus. Since that time, in preparing this substance, I have always added to the usual materials a quantity of potass, either caustic or in the form of sub-carbonate, generally in the proportion of about half a drachm of the latter, to one ounce or one ounce and a half of the former. With this addition much caution in the preparation is not necessary, for it has frequently been made in a pistol barrel and at different temperatures varying from a low red heat to a degree little short of a white. I have never been disappointed in pyrophorus formed in this manner, provided it be transferred as soon as practicable, to a small phial and be excluded from the air. Having had occasion lately to exhibit this compound, a quantity was used which had been prepared six months, and its spontaneous combustion when exposed to the air was equally as vivid as at the time of its formation.—*New Eng. Jour.*

---

Mr. Stevenson will begin his annual Course of Lectures on the Anatomy, Physiology, and Pathology, of the Eye and Ear, early in January, at his house, in Great Russel-street, Bloomsbury.

*Monthly.*

